# Enhancement of Glucose Metabolism via PGC-1α Participates in the Cardioprotection of Chronic Intermittent Hypobaric Hypoxia

**DOI:** 10.3389/fphys.2016.00219

**Published:** 2016-06-08

**Authors:** Xuyi Li, Yan Liu, Huijie Ma, Yue Guan, Yue Cao, Yanming Tian, Yi Zhang

**Affiliations:** ^1^Department of Physiology, Hebei Medical UniversityShijiazhuang, China; ^2^Hebei Collaborative Innovation Center for Cardio-Cerebrovascular DiseaseShijiazhuang, China; ^3^Department of Endocrinology, The Third Hospital of Hebei Medical UniversityShijiazhuang, China

**Keywords:** chronic intermittent hypobaric hypoxia, cardiac protection, ischemia/reperfusion, glucose metabolism, PGC-1α, rat

## Abstract

**Background and Aims:** Previous studies demonstrated that energy metabolism disturbance impairs cardiac function and chronic intermittent hypobaric hypoxia (CIHH) protects heart against ischemia/reperfusion injury. The present study aimed to test the hypothesis that CIHH protects the heart against ischemia/reperfusion (I/R) injury via improvement of cardiac glucose metabolism.

**Methods:** Male Sprague-Dawley rats received CIHH treatment simulating 5000-m altitude for 28 days, 6 h per day in a hypobaric chamber or no treatment (control). Body weight, fasting blood glucose, blood lipid and glucose tolerance were measured. The left ventricular function of isolated hearts was evaluated during 30 min of ischemia and 60 min of reperfusion using Langendorff method. The mRNA and protein expression involved in cardiac energy metabolism was determined using quantitative PCR and Western blot techniques.

**Results:** 1. There was no difference of body weight, fast blood glucose, blood lipid and glucose tolerance between control and CIHH rats under baseline condition (*p* > 0.05). 2. The recovery of left ventricular function after I/R was improved significantly in CIHH rats compared to control rats (*p* < 0.05). 3. The expression of cardiac GLUT4 and PGC-1α was increased but PDK4 gene expression was decreased by CIHH treatment at both mRNA and protein level. Also p-AMPK/AMPK ratio was increased in CIHH rats (*p* < 0.05).

**Conclusion:** CIHH ameliorates I/R injury through improving cardiac glucose metabolism via upregulation of GLUT4, p-AMPK, and PGC-1α expressions, but downregulation of cardiacPDK4 expression.

## Introduction

It is well known that cardiovascular disease is the leading cause of mortality, morbidity, and health care costs globally (Fernando et al., 2015). To date, only limited success has been achieved in preventing and treating ischemic heart diseases (Bu et al., 2015). The normal structure and function of the heart depend on stabilization of energy metabolism. Disturbed energy supply or altered energy substrate utilization has been found to be closely correlated with cardiac patho-physiological conditions, leading to impaired cardiac function, such as increased susceptibility to ischemia-reperfusion (I/R) injury or impaired function recovery after I/R (Anzawa et al., 2007; Harmancey et al., 2013). Therefore, optimization of cardiac energy metabolism provides a strategy to protect against ischemic heart disease.

It has been shown that chronic intermittent hypobaric hypoxia (CIHH), similar to ischemic preconditioning and high altitude hypoxia adaptation, confers cardiac protection against ischemia/reperfusion (I/R) injury in rats (Zhou et al., 2013, 2015; Ma et al., 2014; Bu et al., 2015). The CIHH-induced cardiac protection persists longer than ischemic preconditioning and is associated with fewer side effects, such as polycythemia, right ventricular hypertrophy and pulmonary hypertension compared with long-term adaptation to high-altitude hypoxia (Neckar et al., 2004; Zhang and Zhou, 2012; Bu et al., 2015). Multiple mechanisms and pathways may be involved in the cardio-protective effect of CIHH, such as increased activity and expression of antioxidative enzyme (Balkova et al., 2011), activation of an ATP-sensitive potassium channel and inhibition of the mitochondrial permeability transition pore opening (Bu et al., 2015), increase in myocardial capillary density and coronary blood flow (Zhong et al., 2002), attenuation of beta-adrenergic receptor activity (Guan et al., 2010), induction of heat shock protein 70 expression (Zhong et al., 2000), activation of protein kinase C, and enhancement of resistance against calcium overload (Chen et al., 2013; Ma et al., 2014).

Although abundant data are available concerning the effects of CIHH on the myocardium, the research on CIHH is more concentrated on heart hemodynamics, electrophysiology, and cell apoptosis, etc. The mechanisms of its protective effect, especially the mechanisms of energy metabolism, have not yet been clearly elucidated. The aim of present study is to investigate if glucose metabolism participates in the cardiac protection of CIHH by using functional, morphological, biochemical, and molecular biological methods.

## Methods

### CIHH treatment

All animal experiments were conducted in compliance with the Guide for the Care and Use of Laboratory Animals (Publication 85–23, revised 1996; National Institutes of Health, Bethesda, MD), and all techniques and procedures were reviewed and approved by Hebei Medical University Institutional Animal Care and Use Committee. Age- and body-weight-matched male Sprague–Dawley rats (supplied by Hebei Medical University Animal Center) were randomly divided into control group and CIHH group (subjected to a hypobaric hypoxia mimicking 5000-m altitude for 28 days, 6 h per day in a hypobaric chamber where PB = 404 mmHg, PO_2_ = 84 mmHg). The control rats were housed in same environment as CIHH rats but without CIHH exposure. All animals had free access to water and a standard laboratory diet and were housed in a temperature-controlled room (22 ± 1°C) with a 12 h/12 h light/dark cycle. Body weight of rats was collected each week.

### Blood glucose and blood lipid determination

Rats were fasted overnight and were sacrificed the next day between 9:00 and 12:00 a.m. after blood samples were collected from the abdominal artery under anesthesia (*n* = 6 per group). Blood glucose was measured by glucose meter (ROCHE, Germany) immediately after blood sampling. The plasma was separated by centrifuge and stored at −20°C until analysis. Plasma concentrations of total cholesterol, triglycerides and non-esterified fatty acids were measured with commercially available kits.

### Glucose tolerance test

Animals were fasted overnight and then intraperitoneally injected with glucose (50% solution; 2 g/kg body weight). Blood samples were taken at 0- (before injection), 30-, 60-, and 120-min after glucose injection. Plasma glucose was determined as described above. Glucose tolerance was assessed with the area under the blood glucose curve (AUC), which was calculated according to the equation below:

AUC = (blood glucose at 0-min + blood glucose at 30-min) × 0.25 + (blood glucose at 30-min + blood glucose at 60-min) × 0.25 + (blood glucose at 60-min + blood glucose at 120-min) × 0.5.

### Measurement of ventricular functions in isolated rat hearts

The rats were anesthetized with sodium pentobarbital (50 mg/kg) and their hearts were quickly removed and placed in ice-cold Krebs-Henseleit buffer containing (in mmol/L): NaCl 118.0, KCl 4.7, CaCl_2_ 2.5, MgSO_4_ 1.2, NaHCO_3_ 25.0, KH_2_PO_4_ 1.2, and glucose 11.0 (gassed with 95% O_2_ and 5% CO_2_, pH adjusted to 7.4). Then the hearts were retrogradely perfused via the aorta with Krebs-Henseleit buffer at constant pressure (10 kPa) and temperature (37°C) on a Langendorff apparatus (Chengdu Instrument, Sichuan, China). A water-filled latex balloon-tipped catheter was placed into the left ventricle through the left atrium and adjusted to a left ventricular end diastolic pressure (LVEDP) of 3–10 mmHg during the initial equilibration. The distal end of the catheter was connected to a PowerLab system via a pressure transducer (MLT0380/D, AD Instrument Ltd., Australia). After 20 min stabilization with K-H buffer, the hearts subjected to 30 min no-flow global ischemia followed by 60 min reperfusion. For myocardial infarction determination, the reperfusion time for the heart was prolonged to 120 min. Left ventricular developed pressure (LVDP), LVEDP, maximum rate of rise of left ventricular developed pressure (+LVdp/dt_max_), and maximum rate of decline of left ventricular developed pressure (−LVdp/dt_max_) were continuously recorded by PowerLab system (AD Instrument Ltd, Australia). The data were analyzed by Chart software (AD Instrument, Australia).

### Determination of myocardial infarct size

The isolated rat heart was subjected to 30 min of ischemia and then followed by 120 min of reperfusion. At the end of reperfusion, the heart was removed quickly and frozen in −20°C. The frozen heart was cut into about 1.5 mm thin slices, which were perpendicular to the septum from the apex to the base. Then the slices were incubated in sodium phosphate buffer containing 1% (wt/vol) 2,3,5-triphenyl tetrazolium chloride (TTC) for 10 min to visualize the unstained infarct region. The infarct myocardium that was not stained by TTC looked pale. Microphotography was taken with a digital camera. The image processing system (Motic MED 6.0, Xiamen, China) was used to analysis the data by setting a reference value, and the software could automatically select the similar region for the infarct size. The extent of infarct myocardium was expressed as the percentage of the infarct size to the ventricular size.

### RNA isolation and quantitative real-time PCR

Heart tissues used for RNA isolation were rapidly excised and frozen in liquid nitrogen. RNA was extracted from heart samples by using TRIzol reagent (Invitrogen, USA) method following manufactory's protocol. The integrity of total RNA was assessed using Nano Drop 2000 Spectrophotometer (Thermo, USA). Total RNA was reverse transcribed by Invitrogen 2-step RT kits Superscript II first strand synthesis kit (Invitrogen, USA) according to the manufacturer's instructions.

The cDNA was 1:20 diluted and used for real-time PCR. Platinum SYBR Green qPCR Super Mix-UDG with ROX kits (Invitrogen, USA) were used to measure gene expression using specific primer sets: *pdk4, glut4, ampk, pgc-1*α, and *gapdh* (Invitrogen, USA) (Table 1). Dissociation curves were run following Real-Time PCR reactions to ensure the detection of the desired amplicon and exclude the presence of contaminating products. All reactions were performed in the ABI Prism 7000 Sequence Detection System (Applied Biosystems, USA). Gene expression was normalized to *gapdh* and the data were analyzed using comparative 2^∧^−ΔΔCt method.

**Table 1:** **The sequences of primers used for Real-time PCR**.

**Gene**	**Forward primer**	**Reverse primer**
*ampk*	5-TCCCGTGTTTACAGATGTAGTCG-3	5-AAGATTCGCAGTTTAGATGTTGTTG-3
*gapdh*	5-GGACCAGGTTGTCTCCTGTG-3	5-ATTCGAGAGAAGGGAGGGCT-3
*glut4*	5-TCAAAGATGCTGGTTGAATAGTAGAA-3	5-GATGAGAAACGGAAGTTGGAAAG-3
*pdk4*	5-CGACAGATTCTTTGGTTCCTTACTT-3	5-CCCCTTTGGCTGGTTTTGGTTAC-3
*pgc-1α*	5-GAATGCAGCGCTCTTAGC-3	5-GCTTTTGCTGTTGACAAATG-3

### Western blot

Heart tissues used for western blot were rapidly excised and frozen in liquid nitrogen. Proteins from heart tissue were extracted in lysis buffer containing 50 mM Tris-HCl (pH: 8.0) and complete protease inhibitor cocktail tablet. After protein quantification, fifty micrograms of total protein extracts from rat heart tissue were electrophoresed through polyacrylamide SDS gels and transferred by electroblotting onto PVDF membranes. Membranes were blocked for 1 h in 5% (w/v) nonfat milk before incubation with appropriate dilutions of antibodies of PDK4 (1:1500, Proteintech, USA), CPT1B (1:1000, Aviva Systems Biology, USA), AMPKα1 (1:1000, Empitomics, USA), Phospho-AMPK alpha (Thr 172) (1:1000, Affbiotech, USA), PGC-1α (1:100, Biorbyt, UK), GLUT4 (1:1000, Empitomics, USA), GAPDH (1:5000, Empitomics, USA) at 4°C overnight and then with corresponding secondary antibodies (1:5000). The blots were developed using the ECL system (immobilion TM Western, Millipore) and were analyzed by Quantity One Software (Bio-Rad, USA). Protein contents were normalized by glyceraldehyde-3-phosphate dehydrogenase (GAPDH, Abcam, USA) level.

### Data analysis

Data were expressed as mean ± SD. Student-Newman-Keuls *T*-test was used for comparison between two groups. One-way ANOVA followed by *SNK* test were used for multiple comparisons. *P* < 0.05 was considered statistically significant.

## Results

### The effect of CIHH on body weight, glucose concentration, lipid concentration, and glucose tolerance test

**Figure 1:**
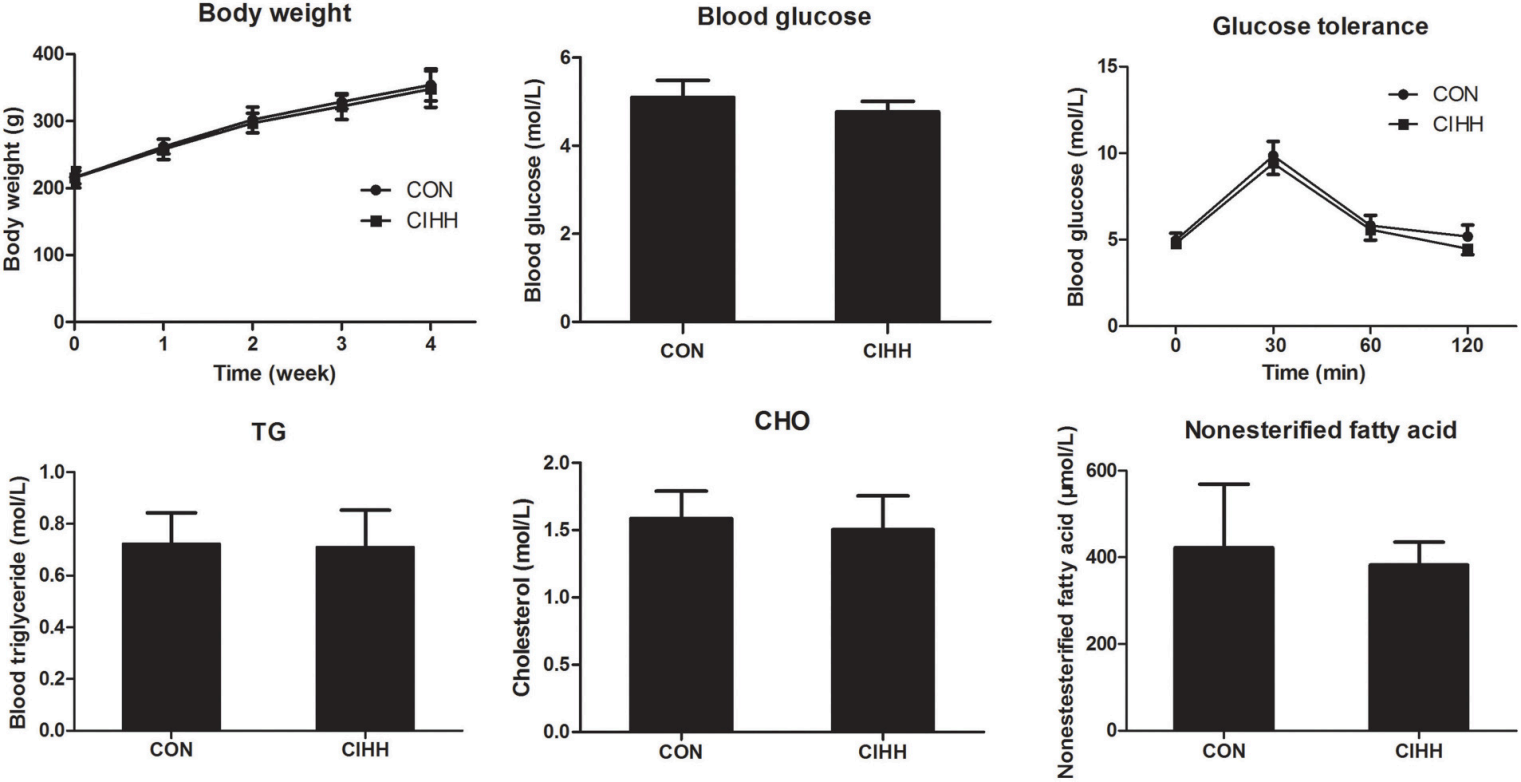
**Effects of CIHH on body weight, blood glucose, Triglyceride (TG), Cholesterol (CHO), nonesterified fatty acid, and glucose tolerance in rats**. Data are expressed as the mean ± SD (*n* = 6 for each group).

There was no difference of body weight, fast blood glucose and glucose tolerance between control and CIHH rats (*P* >0.05). There was no significant difference of blood TG, TC and nonesterified fatty acid between CON and CIHH rats (*P* > 0.05, Figure 1).

### The effects of CIHH on ventricular function and cardiac infarct size

**Figure 2:**
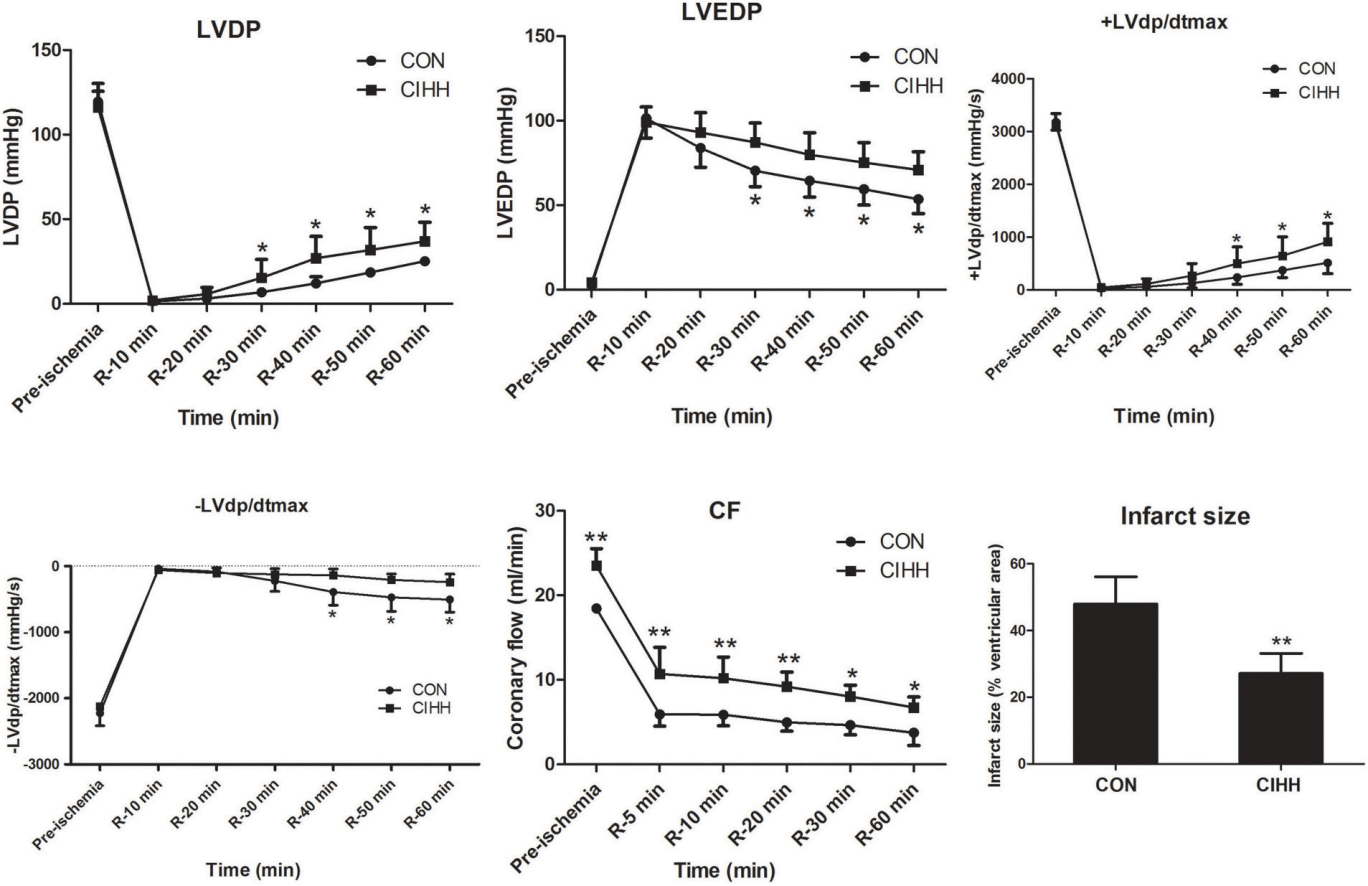
**Effect of chronic intermittent hypobaric hypoxia on cardiac function and infarct size in isolated rat hearts subjected to 30 min of global ischemia followed by 60 min of reperfusion**. LVDP, Left ventricular developing pressure; LVEDP, left ventricular end-diastolic pressure; +LVdp/dt_max,_ maximum rate of rise of left ventricular developed pressure; −LVdp/dt_max,_ maximum rate of decline of left ventricular developed pressure; CF, coronary flow. Data are expressed as the mean ± SD (*n* = 6 for each group). ^*^P < 0.05, ^**^P < 0.01 *vs*. corresponding CON group.

Under baseline conditions, the coronary flow (CF) was significantly higher in CIHH rats than in CON rats. However, the basal left ventricular functions indicated by LVDP, ±LVdp/dt_max_, and LVEDP did not differ between CON and CIHH rats. The LVDP and ±LVdp/dt_max_ were decreased, whereas LVEDP was increased during I/R, suggesting the left ventricular function was impaired by I/R. However, the recovery of cardiac function and CF after I/R was improved in CIHH rats compared with control rats (*P* < 0.05 or *P* < 0.01, Figure 2). The myocardial infarct size of CIHH rats was also decreased compared with CON rats (*P* < 0.01, Figure 2).

### The effects of CIHH on mRNA expression involved in cardiac energy metabolism

**Figure 3:**
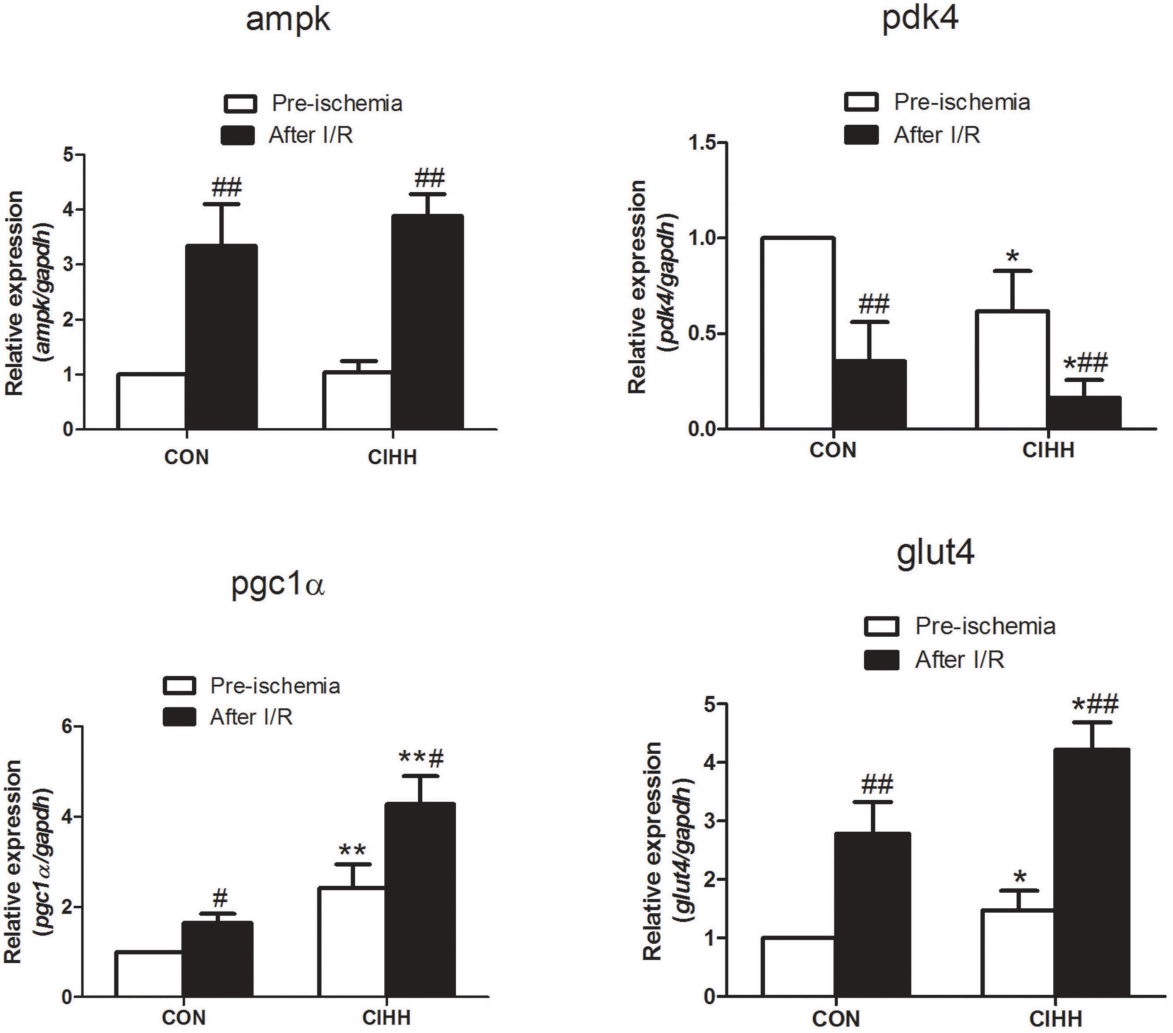
**Effect of CIHH on gene expression of cardiac ***ampk, pdk4, glut4***, and ***pgc-1***α using Realtime PCR**. Data are expressed as the mean ± SD (*n* = 6 in each group). ^*^P < 0.05, ^**^P < 0.01 *vs*. corresponding CON group; ^#^*P* < 0.05, ^*##*^*P* < 0.01 *vs*. corresponding pre-ischemia.

To further determine the mechanisms responsible for the protective effect of CIHH on myocardial energy metabolism during I/R, gene expression involved in cardiac energy metabolism was determined using quantitative PCR. After I/R, the mRNA expression of *ampk, pgc-1*α and *glut4* in myocardium was increased, while pdk4 was decreased in both CIHH and control rats. CIHH increased cardiac *pgc-1*α, and *glut4*, but decreased *pdk4* mRNA expression more significantly than CON group (*P* < 0.05, Figure 3). Meanwhile, *ampk* mRNA expression not differ between the two groups (*P* > 0.05, Figure 3).

### The effects of CIHH on protein expression involved in cardiac energy metabolism

**Figure 4:**
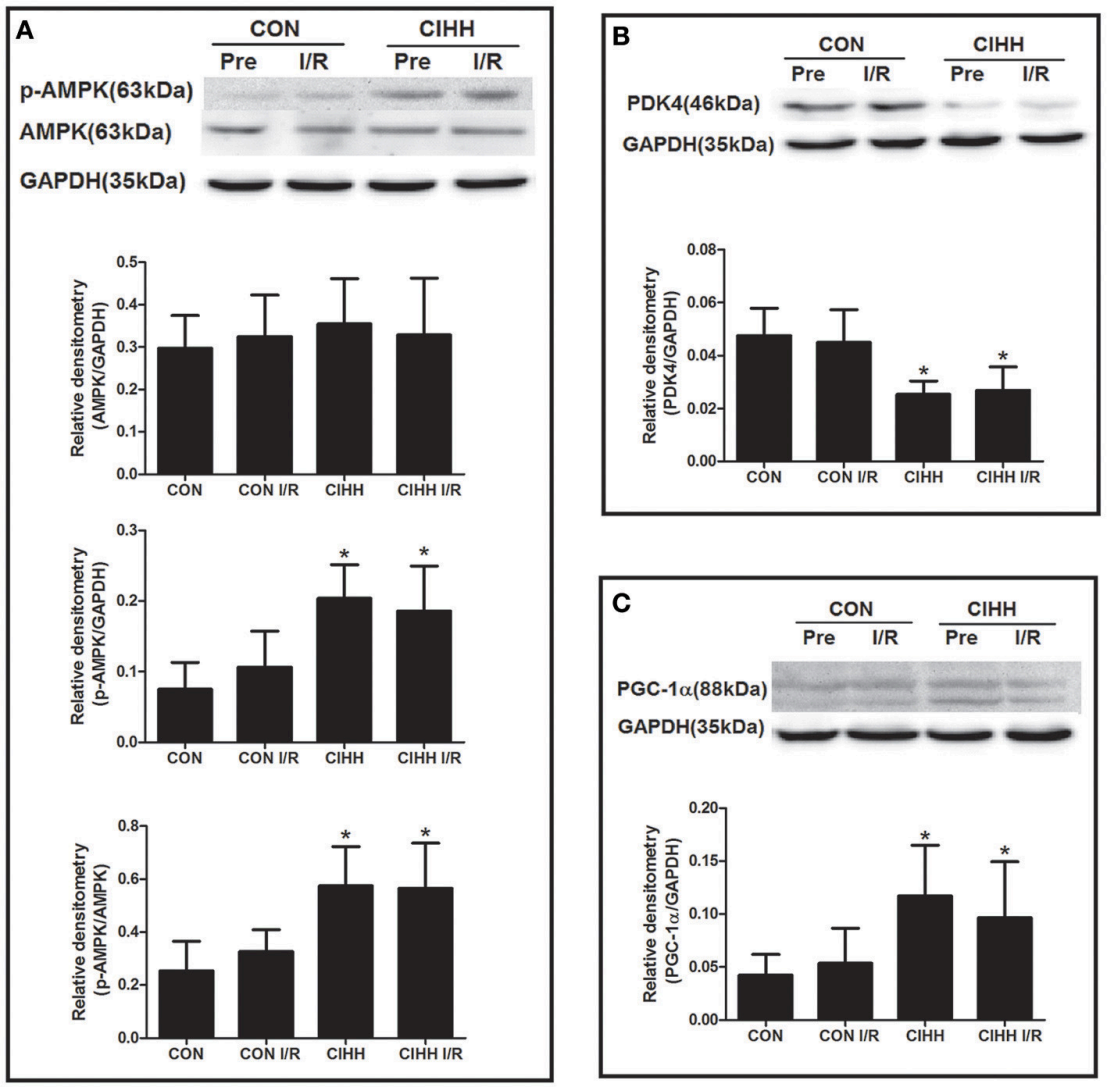
**Effect of CIHH on the protein expression of AMPK, p-AMPK, PDK4 and PGC-1α in left ventricular myocytes before and after I/R. (A)** Protein expression of AMPK and p-AMPK; **(B)** Protein expression of PDK4; **(C)** Protein expression of PGC-1α. CON, control group; CIHH, chronic intermittent hypobaric hypoxia; Pre, pre-ischemia; I/R, ischemia/reperfusion. Data are expressed as the mean ± SD (*n* = 6 in each group). ^*^P < 0.05 *vs*. corresponding CON group.

**Figure 5:**
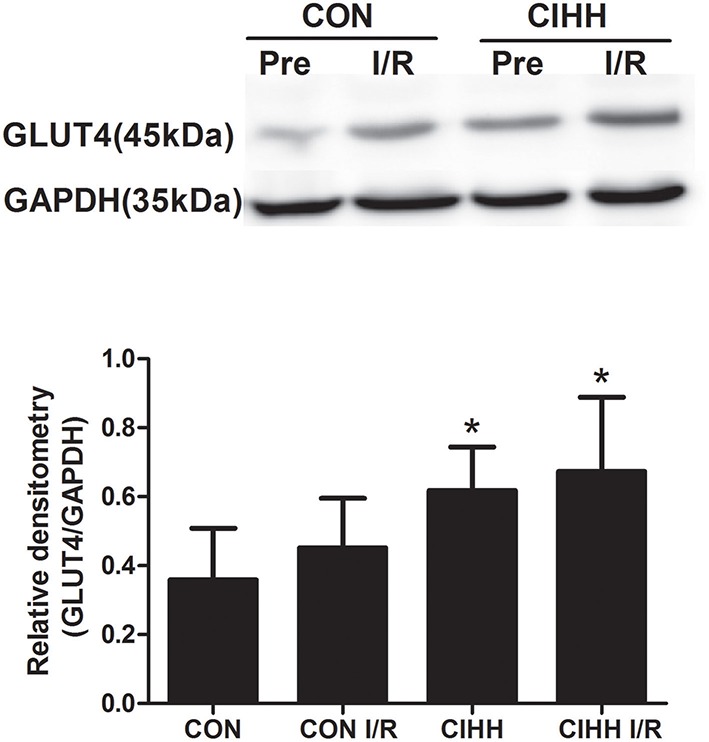
**Effect of CIHH on the protein expression of GLUT4 in left ventricular myocytes before and after I/R**. CON, control group; CIHH, chronic intermittent hypobaric hypoxia; Pre, pre-ischemia; I/R, ischemia/reperfusion. Data are shown as expressed as the mean ± SD (*n* = 6 in each group). ^*^P < 0.05 *vs*. corresponding CON group.

Protein expression involved in cardiac energy metabolism was determined using Western blot. The results showed that CIHH significantly increased cardiac p-AMPK/AMPK ratios, PGC-1α, and GLUT4 expression, but decreased PDK4 protein expression compared to CON group (*P* < 0.05, Figures 4, 5). There was no difference of AMPK protein expression between two groups (*P* > 0.05, Figure 4). Furthermore, there were no differences of AMPK, PDK4, PGC-1α, and GLUT4 expression between before and after I/R in both CON and CIHH groups (*P* > 0.05, Figures 4, 5).

## Discussion

In this study, we investigated the role of energy metabolism in cardiac protection of CIHH. The results showed that there was no difference between control and CIHH rats in body weight, fasting blood glucose, blood lipid, and glucose tolerance under basic condition. Consistent with previous studies (Zhang et al., 2007), we confirmed again that CIHH improves the recovery of cardiac function after I/R. Furthermore, CIHH significantly increased the expression of cardiac PGC-1α and GLUT4, but decreased PDK4 at both mRNA and protein level. In addition, CIHH significantly increased the ratio of p-AMPK/AMPK protein expression. We demonstrated for the first time that CIHH ameliorates cardiac I/R-induced injury by increasing GLUT4, p-AMPK, and PGC-1α expression, but reducing PDK4 expression in cardiomyocytes.

The constant workload of the heart requires a continuous energy supply in the form of adenosine triphosphate (ATP) to maintain its contractile function. However, the myocardial storage of ATP is insufficient, and must be synthesized continuously. ATP may come from a variety of energy substrate, such as fatty acids, sugars and metabolic products such as lactic acid, pyruvic acid and ketone. Among the myocardial substrates, glucose accounts for less than 25% of the energy production under normal conditions. However, glucose is unique among myocardial substrates because a small amount of ATP is obtained by substrate-level phosphorylation during glycolysis even in situations of hypoxia or ischemia. ATP obtained from glycolysis in the extramitochondrial compartment, although scarce, may be of paramount importance for the maintenance or restoration of ionic homeostasis (Montessuit and Lerch, 2013). It has been found that diabetic heart, characterized by reduced glucose oxidation, is more prone to I/R injury (Anzawa et al., 2007; Harmancey et al., 2013). Cardiac function recovery after I/R was improved by partial stimulation of glucose oxidation (Gandhi et al., 2008; Lucchinetti et al., 2011; Nagendran et al., 2013). Therefore, improvement of myocardial cell glucose metabolism could increase the heart's tolerance to ischemic injury (Finck and Kelly, 2007; Li et al., 2010; Rowe et al., 2010).

The first step of glucose metabolism is the transport of glucose across the plasma membrane. Glucose transport occurs by facilitated diffusion through selective transport proteins of the GLUT family. In cardiomyocytes, mostly two isoforms of glucose transporter, GLUT1 and GLUT4, are involved. GLUT1 predominates during fetal and early postnatal life (Montessuit and Lerch, 2013). GLUT4, on the other hand, is the main isoform presented in fully differentiated cardiomyocytes. GLUT4 has a higher affinity for glucose than GLUT1, which makes GLUT4 responsible for the majority of glucose uptake (Montessuit and Lerch, 2013). It was reported that augmented glucose uptake by GLUT4 displayed a protective effects during cardiac ischemia in neonatal or adult mice (Shi et al., 2016) and normal or streptozotocin (STZ)-treated diabetic rats (Ji et al., 2013). Our study showed that CIHH increased cardiac GLUT4 expression at both mRNA and protein levels, indicating that CIHH may improve glucose metabolism through increase of cardiac glucose uptake.

Before glucose enters the oxidative pathways of the mitochondrion, it must first undergo anaerobic metabolism in the cytosol of the cardiac myocyte and be converted to pyruvate. Then pyruvate can be converted to lactate outside the mitochondrion or oxidized in the mitochondrial matrix to generate acetyl-CoA by the action of pyruvate dehydrogenase (PDH) for the tricarboxylic acid cycle (Finck and Kelly, 2007) and ATP was produced. PDH, a rate-limiting enzyme in glucose oxidation, is inhibited by PDH kinase-4 (PDK-4). PDH inhibition leads to reduction of glucose oxidation and diversion of glycolytic intermediates to alternative metabolic pathways. Some studies displayed that decrease of PDK4 could improve the glucose metabolism (Gao et al., 2014, 2015) and contribute to protection against I/R injury. In the present study, CIHH reduced PDK4 expression at both mRNA and protein levels, indicating that CIHH may improve glucose metabolism through increase of cardiac glucose oxidation by decrease of PDK-4.

PGC-1s proteins, including PGC-1α, PGC-1β, and PGC-1–related coactivator, enhance the transcriptional activity of transcription factors through direct protein-protein interactions. PGC-1α is highly inducible in response to increased demand for myocardial ATP productionand acts as an important energy regulator in the heart (Lu et al., 2010; Leone and Kelly, 2011). PGC-1α gene expression is also sensitive to metabolic sensors such as adenosine monophosphate-activated protein kinase (AMPK). The expression of PGC-1α is repressed in numerous models of heart failure, and has been indicated as an important contributor to the maladaptive energetic profile of failing hearts (Rowe et al., 2010). Mice lacking PGC-1α develop signatures of heart failure with a marked drop in cellular ATP concentration. The expression of PGC-1α and its targets is downregulated in pathological cardiac hypertrophy and failing heart (Finck and Kelly, 2007). However, moderate overexpression of PGC-1α contributes to the protective effect of cardiomyocytes (Rowe et al., 2010; Liu et al., 2015), which could be elucidated by the regulation of the glucose uptake and oxidation (Finck and Kelly, 2006). Our study showed that CIHH increased the expression of PGC-1α both mRNA and protein, suggesting PGC-1α could improve the glucose uptake and oxidation, which may improve the energy metabolism of the cardiomyocytes and reduce the I/R injury in rat heart.

AMPK is the key factor in the heart that orchestrating the cellular response to a variety of stresses and regulates metabolism and organelle function. Recent investigations have shed light on the cardio-protective role of AMPK in I/R injury as well as in pathological hypertrophy and failure (Gundewar et al., 2009; Gauthier et al., 2011; Kim and Tian, 2011). AMPK can be activated by phosphorylation of the Thr172 site, sensing the fuel shift and then phosphorylate and activate downstream targeting genes such as FoxO and PGC-1α (Jager et al., 2007; Greer et al., 2009). Therefore, increased expression of AMPK, especially phosphorylated AMPK (p-AMPK) may contribute to protecting the heart against I/R injury. In our study, CIHH did not affect the expression of AMPK, but the protein expression of p-AMPK and p-AMPK/AMPK ratios increased. The results indicate that CIHH enhances the activation of AMPK, which contributes to the cardiac protection against I/R injury.

In conclusion, this study demonstrated for the first time that CIHH protects the heart against I/R injury through improvement of the energy metabolism via upregulating cardiac GLUT4, p-AMPK, and PGC-1α expressions and downregulating cardiac PDK4 expression, which will provide a new concept or strategy for prevention and treatment of ischemic heart disease.

## Author contributions

Designed the experiments: HM; performed the experiments: XL, YG, YC, YT; analyzed the data: YL; wrote the manuscript: XL; revised the manuscript: HM, YZ.

### Conflict of interest statement

The authors declare that the research was conducted in the absence of any commercial or financial relationships that could be construed as a potential conflict of interest.
